# The Use of a Chronic Disease and Risk Factor Surveillance System to Determine the Age, Period and Cohort Effects on the Prevalence of Obesity and Diabetes in South Australian Adults - 2003–2013

**DOI:** 10.1371/journal.pone.0125233

**Published:** 2015-04-29

**Authors:** Anne W. Taylor, Zumin Shi, Alicia Montgomerie, Eleonora Dal Grande, Stefano Campostrini

**Affiliations:** 1 Population Research & Outcome Studies, Discipline of Medicine, The University of Adelaide, South Australia, Australia; 2 Ca’ Foscari University, Venice, Italy; Sun Yat-sen University, CHINA

## Abstract

**Background:**

Age, period and cohort (APC) analyses, using representative, population-based descriptive data, provide additional understanding behind increased prevalence rates.

**Methods:**

Data on obesity and diabetes from the South Australian (SA) monthly chronic disease and risk factor surveillance system from July 2002 to December 2013 (n = 59,025) were used. Age was the self-reported age of the respondent at the time of the interview. Period was the year of the interview and cohort was age subtracted from the survey year. Cohort years were 1905 to 1995. All variables were treated as continuous. The age-sex standardised prevalence for obesity and diabetes was calculated using the Australia 2011 census. The APC models were constructed with ‘‘apcfit’’ in Stata.

**Results:**

The age-sex standardised prevalence of obesity and diabetes increased in 2002-2013 from 18.6% to 24.1% and from 6.2% to 7.9%. The peak age for obesity was approximately 70 years with a steady increasing rate from 20 to 70 years of age. The peak age for diabetes was approximately 80 years. There were strong cohort effects and no period effects for both obesity and diabetes. The magnitude of the cohort effect is much more pronounced for obesity than for diabetes.

**Conclusion:**

The APC analyses showed a higher than expected peak age for both obesity and diabetes, strong cohort effects with an acceleration of risk after 1960s for obesity and after 1940s for diabetes, and no period effects. By simultaneously considering the effects of age, period and cohort we have provided additional evidence for effective public health interventions.

## Background

The prevalence rates of both obesity and type 2 diabetes have increased in recent decades in most western countries [1–4]. Although there are many reasons cited for these increases, including economic transformations and lifestyle changes such as increased food consumption and decreased physical activity, many of the important factors remain relatively uncertain [1,5]. Age, period and cohort (APC) analyses, using representative, population-based descriptive data, provide additional understanding behind increased prevalence rates [6]. The APC variables of age, period and cohort, although all related in someway to time, take into account a plethora of individual, societal, historical and cultural aspects associated with life [7].

Age effects are designed to reflect developmental, biological and life-course changes linked with the physiological changes associated with ageing [7,8]. Increased age is associated with escalated chronic conditions including diabetes. Obesity is also more prevalent in middle-age and older populations and has been related to increased all-cause and cause-specific mortality in these age-groups [9].

Period effects are associated with changes in the social, economic, cultural or physical environment that affect the whole population at the same time and result in whole populations changing habits simultaneously [7,8,10]. In terms of diabetes and obesity they take into account major historical events that have occurred (economic or policy changes) in the period. These include, for the examples of diabetes and obesity, increased production of energy-dense food, decreased physical activity at work and home caused in part by increased technical innovation [11], increase in sugar laden soft-drink consumption [5], and economic conditions such as the boom and recession periods.

Cohort effects are seen when people, grouped together by their period of birth, experience the same social and historical events during the same period of their lives [5,6]. These unique sets of experiences, that are not the same for people born at different times, include world wars and depressive economic times [11]. Cohort effects are seen when the environment changes, and in the case of obesity and diabetes, lifestyle patterns or behaviours change. For example younger age groups have been born during times of increased technological advances, increased variety of food, less energy expenditure, and increased diet rich in saturated fats while older persons grew up with no TV or computers, and less car travel [12].

The relationship between obesity and type 2 diabetes is well documented with obesity being an important risk factor for the condition [1,4,13–15]. With the recent increases in the proportion of the population who are overweight or obese, a corresponding increase in diabetes prevalence has been shown [1,16]. Using a representative sample of data collected using a chronic disease and risk factor surveillance system over 10 years, we have employed APC methodology to identify and describe patterns and trends and to assist in interpretation of the rise in the prevalence of both obesity and diabetes in South Australia (SA).

## Methods

The data for this analysis were obtained from the SA Monitoring and Surveillance System (SAMSS), a monthly chronic disease and risk factor surveillance system of randomly selected persons, established in July 2002 [17]. All households in SA with a telephone number listed in the Electronic White Pages (EWP) are eligible for selection in the sample. A letter introducing SAMSS is sent to the household of each selected telephone number. Within each household the person who had a birthday last is selected for interview. There is no replacement for non-contactable persons. Data are collected by a contracted agency using Computer Assisted Telephone Interviewing (CATI) and interviews are conducted in English. Detailed SAMSS methodology has been published elsewhere [17]. Ethics clearance was gained from the SA Department of Health and Ageing Human Research Ethics Committee (436.02.2014). Informed consent was gained at the start of the telephone interview. Information was anonymized and de-identified prior to analysis.

SAMSS data collected in the period July 2002 to December 2013 from participants aged 18 years and over (n = 62,674) were used. The monthly response rate of SAMSS for this period was between 60% and 65%. All participants were asked, ‘What is your height without shoes?’ and ‘What is your weight?’ (Undressed in the morning). Body Mass Index (BMI) was calculated as weight in kilograms divided by the square of height in metres from self-reported current weight and height. BMI was categorised according to the World Health Organisation criteria [18]. Participants with a BMI greater than or equal to 30 kg/m^2^ were classified as obese. Participants were categorised as having diabetes if they reported being ever been told by a doctor that they had diabetes.

Age was the self-reported age of the respondent at the time of the interview. Period was the year of the interview and cohort was calculated by subtracting age from the survey year. Cohort years range from 1905 to 1995. All variables were treated as continuous.

SAMSS data were weighted each month by age, sex, area and probability of selection in the household to estimated resident population data to the most recent Australian Bureau of Statistics Census or estimated residential population data so that the results were representative of the SA population. Probability of selection in the household was calculated on the number of eligible people in the household and the number of listings in the EWP. The weights reflect unequal sample inclusion probabilities and compensates for differential non-response. We excluded participants with missing data for height and/or weight (n = 3649). This yielded a final study size of n = 59025.

SPSS Version 19 was used for descriptive statistics including prevalence, means and medians. Stata Version 13.0 was used for the age-sex standardised prevalence for obesity and diabetes and was calculated using the Australia 2011 Census. The APC models were constructed with ‘‘apcfit” in Stata that uses natural splines to estimate each of the three effects that are then combined to give estimated rates [19,20]. We used five knots each for age, period, and cohort variables to detect non-linear effects, with 1950 as the cohort referent and 2007 as the period referent group.

## Results

**Fig 1:**
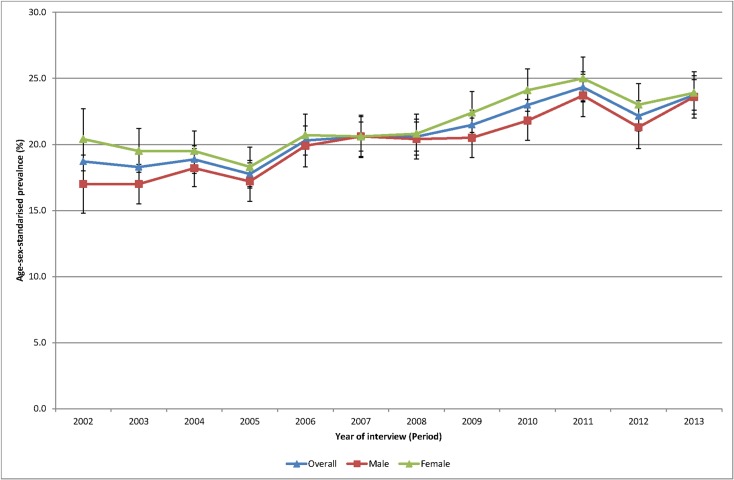
Age sex standardised prevalence of obesity, 2002 to 2013.

**Fig 2:**
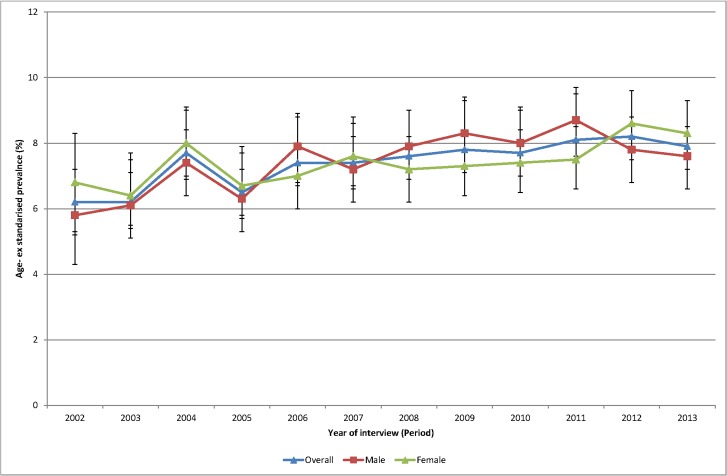
Age sex standardised prevalence of diabetes, 2002 to 2013.

Of the total sample 49.7% were male. Mean age was 47.6 years (median 46.0 years) and ranged from 46.5 years (median 45) in 2003 to 48.8 years (median 49 years) in 2013. The age-sex standardised prevalence of self-reported obesity (Fig 1) increased during the period 2002–2013 from 18.6% in 2002 to 24.1% in 2013. The age-sex standardised prevalence of diabetes (Fig 2) increased during the period 2002–2013 from 6.2% to 7.9%.

**Fig 3:**
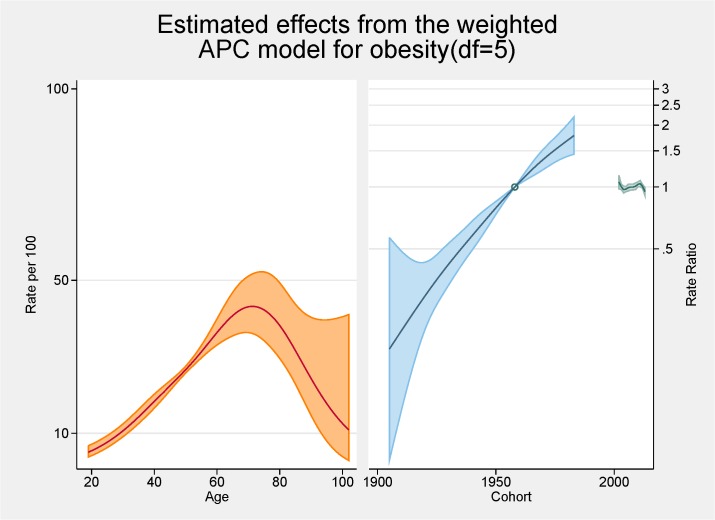
Multivariable APC-analysis of obesity per 100 2002–2013, shown as line graphs representing rates (%) and rate ratios (log scale), with its 95% confidence interval (shaded area). The left line is the estimated age effect, the middle line refers to the estimated birth cohort effect and the short line to the right refers to the estimated period effect.

**Fig 4:**
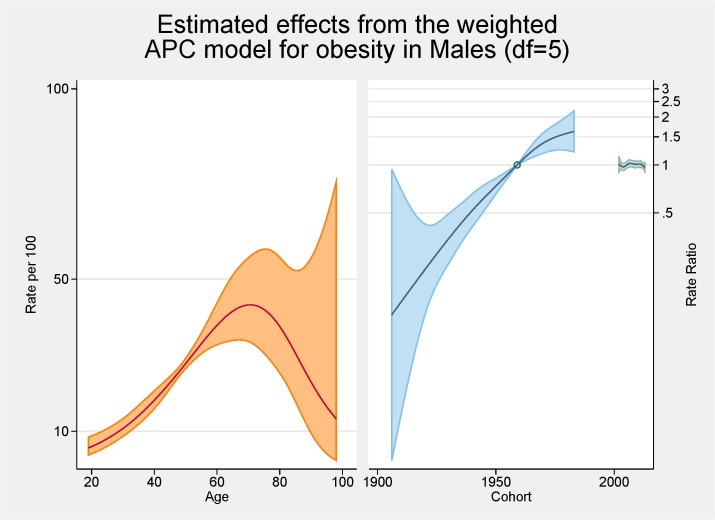
Multivariable APC-analysis of obesity for males per 100 2002–2013, shown as line graphs representing rates (%) and rate ratios (log scale), with its 95% confidence interval (shaded area). The left line is the estimated age effect, the middle line refers to the estimated birth cohort effect and the short line to the right refers to the estimated period effect.

**Fig 5:**
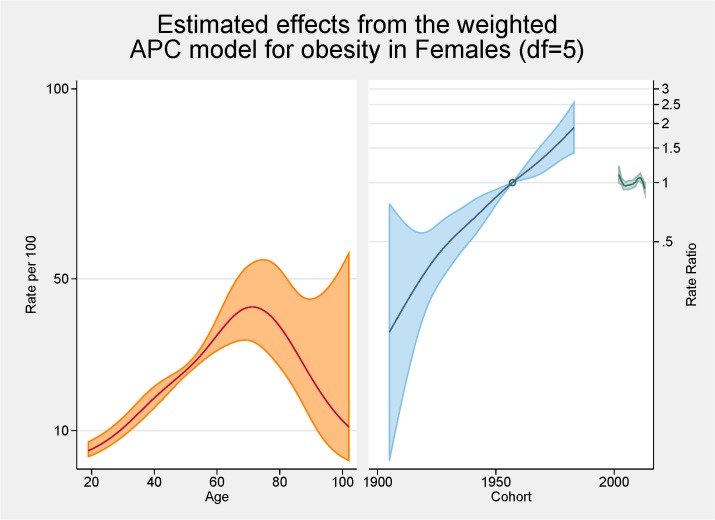
Multivariable APC-analysis of obesity for females per 100 2002–2013, shown as line graphs representing rates (%) and rate ratios (log scale), with its 95% confidence interval (shaded area). The left line is the estimated age effect, the middle line refers to the estimated birth cohort effect and the short line to the right refers to the estimated period effect.

Fig 3 shows the fitted model for the total population with the independent effects of age (proportion of overall obesity), period and cohort (both as the rate ratios for overall obesity). The peak age for obesity is approximately 70 years with a steady increasing rate from 20 to 70 years of age. There are strong cohort effects and no period effects. Figs 4 and 5 show the same fitted models for males and females separately.

**Fig 6:**
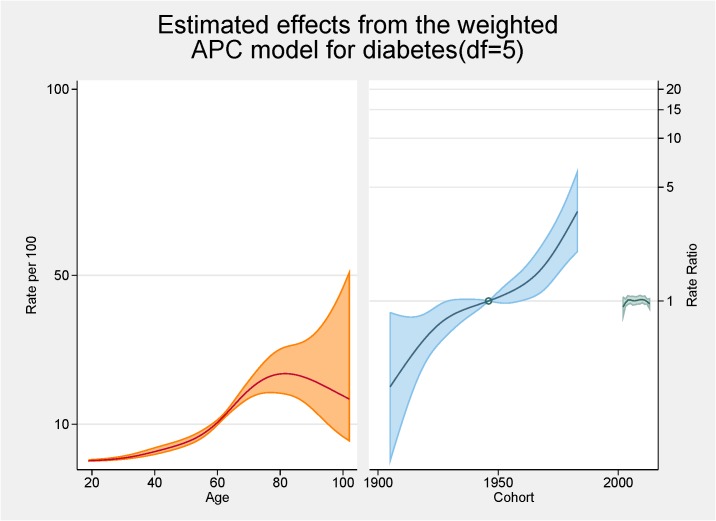
Multivariable APC-analysis of diabetes per 100 2002–2013, shown as line graphs representing rates (%) and rate ratios (log scale), with its 95% confidence interval (shaded area). The left line is the estimated age effect, the middle line refers to the estimated birth cohort effect and the short line to the right refers to the estimated period effect.

**Fig 7:**
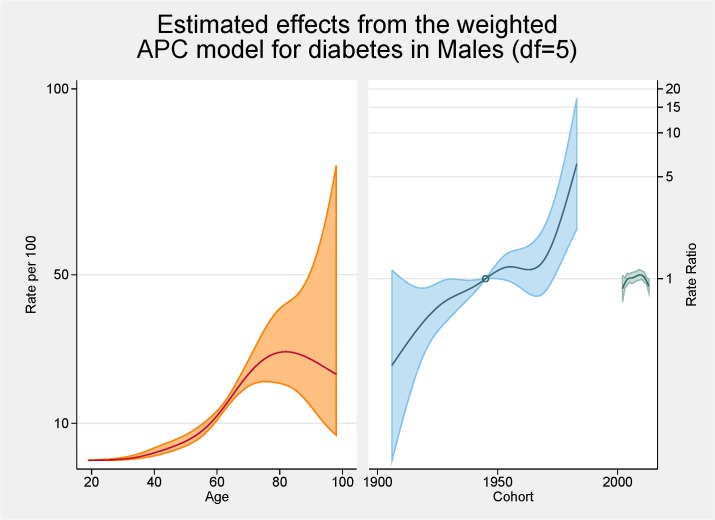
Multivariable APC-analysis of diabetes for males per 100 2002–2013, shown as line graphs representing rates (%) and rate ratios (log scale), with its 95% confidence interval (shaded area). The left line is the estimated age effect, the middle line refers to the estimated birth cohort effect and the short line to the right refers to the estimated period effect.

**Fig 8:**
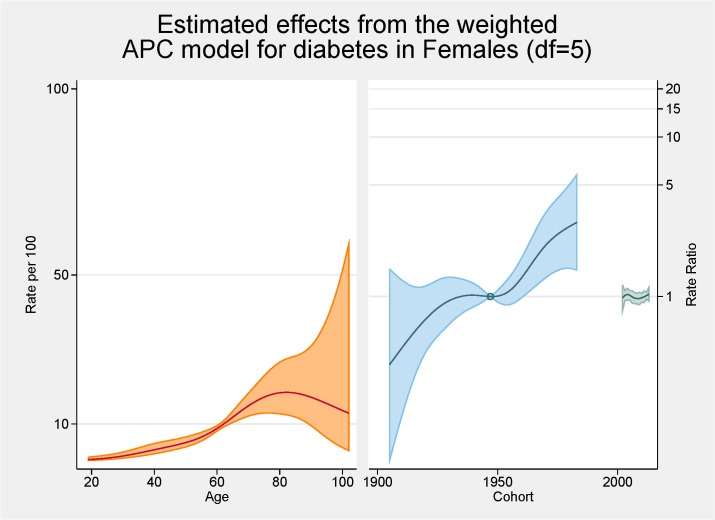
Multivariable APC-analysis of diabetes for females per 100 2002–2013, shown as line graphs representing rates (%) and rate ratios (log scale), with its 95% confidence interval (shaded area). The left line is the estimated age effect, the middle line refers to the estimated birth cohort effect and the short line to the right refers to the estimated period effect.

Fig 6 shows the fitted model for the total population with the independent effects of age (proportion of overall diabetes), period and cohort (both as the rate ratios for overall diabetes). The peak age for diabetes is approximately 80 years. There are strong cohort effects and no period effects. Figs 7 and 8 show the same fitted models for males and females separately.

## Discussion

Initial age-sex standardised prevalence rates over time have shown, in line with most cited analyses, increases for both obesity and diabetes from 2002 to 2013. Females continue to have higher rates of obesity than males although convergence is apparent. Females now also have higher rates of diabetes. The APC analyses showed a higher than expected peak age for both obesity and diabetes, strong cohort effects with an acceleration of risk after 1960s for obesity and after 1940s for diabetes, and no period effects.

From the APC analyses we have shown that the peak age for obesity is approximately 70 years with a steady increasing rate from 20 to 70 years of age. This peak in age is higher than seen in other comparable APC analyses of obesity such as those conducted in Ireland, France and USA with those studies having a peak of obesity prevalence at 59–60 years of age [7,10,21]. Several of the major APC obesity studies had upper age limits or excluded the older age groups because of scarceness of data [11,16,22,23]. A previous Australian study undertaken using 1990–2000 data reported a peak in the late 40s/early 50s with a much flatter plateau effect from the mid 40s to the mid 60s [11]. Our higher than expected peak age could be the result of an increasing proportion of ‘healthy obese’ (those obese but without other risk factors or ill-health) although previous SA work has shown that metabolically healthy obesity was related to younger age groups [24]. Methodologically it could be the result of a healthy interview effect with healthier adults more likely to be interviewed with the SAMSS methodology not interviewing nursing home or institutionalised adults where perhaps more underweight or normal BMI persons might reside. Contrary to these rationales, in a previous study we were able to show that older persons over estimated their current height resulting in a lower BMI which would indicate that BMI could be higher in the older groups [25]. Notwithstanding, it seems that older persons are living longer with higher BMI levels. In this current study there was very little difference between males and females in terms of age with similar underlying trends although the rate of increase was greater for females. This was also seen in the Irish study [10].

This study found there were strong cohort effects, overall, and for males and females with the younger birth cohorts having higher obesity prevalence ratios. There is an obvious acceleration in risk after the 1960s. This has also been shown in an APC analysis in French and USA obesity rates [7,21] although a previous Australian APC analyses showed no obvious cohort effect for obesity although the prevalence rates were higher in younger cohorts. Interestingly the effects for overweight in this previous Australian study mirrored those we found for obesity with marked increases with the cohorts born since 1960s perhaps reflecting the increase in BMI in the population in the last decade [11]. This might also explain some of the age effects discussed in the previous paragraph. In a previous analysis undertaken on another SA population-based survey-based data set, although not using APC methodology, we reported younger generations (born between 1965 and 1980) having the greatest increase in obesity [26]. The effect of technological advances, and cultural and social changes, on this younger cohort is cited as possible reasons for these higher rates [7]. As such, continuation of campaigns aimed at younger persons is warranted.

The cohort effect for obesity is markedly different between the sexes with females having a higher ratio of obesity (nearly 2) in later cohorts. Females also have higher rates earlier but males have a seemingly levelling off in later cohorts while for females no such plateauing is evident. In the French APC analysis there was also a stabilisation for cohorts born in 1970s and 1980s [21]. While our overall results show no such trend there is an indication males may be stabilising in these later cohorts. Robinson et al also showed different cohort effects for males and females in their APC assessment of abdominal obesity in the USA [22]. Robinson et al’s rational for the increased cohort rates for females centred on the major social changes experienced by females in the 20^th^ century including fertility, work physical activity levels, and economic changes [22].

No period effects were evident in our study. This is contrast to the Irish study, which reported positive period effects citing negative changes in dietary patterns and physical activity levels for these increases [10]. Our lack of period effects is perhaps showing a halting of the increase in the obesity prevalence in contrast to the previous Australian work that showed clear period effects with increases at each of the surveys, especially for females [11]. Similar to our findings, relatively recent NHANES data reported a peak of obesity rates for females [27]. Reither et al argues that period effects and the resultant changes in society are at ‘the root of the obesity epidemic’ [27]. They argue factors such as less physical activity at work and higher consumption of calorie risk food have considerably influenced period effects.

In terms of diabetes, the relationship between advancing age and diabetes is well known [14]. The peak in our analyses is approximately 80 year of age and this, similar to the obesity trends, is higher than normally cited [28]. Similar reasons to the higher rates for obesity are possible. The pattern differs by gender with an increased proportion of males having diabetes younger than females; at age 60 approximately 12% of males have diabetes while this figure is <10% for females. This has also been shown in other Australian studies [28].

In terms of cohort effects for diabetes there is a marked acceleration after 1940s. This was evident for both males and females although by the end of the study period the ratio for males was over six while for females the increase was markedly less. Unexplained is the dip in the cohort effect for males around the mid 1960s. Cohort effects are often the impact of nutrition in pregnancy or early childhood [29] but why this is evident for males only is unresolved. Kwon also reported an increase trend in diabetes for younger cohorts in his APC analysis of Korean men [16].

No period effects for diabetes were seen. There are very few APC analyses cited in the literature so comparison with other studies are limited although Kwon et al also reported a period effect in their study of men aged 20 to 59 years [16].

Comparing the APC analysis for obesity and diabetes, it is apparent that the magnitude of the cohort effect is much more pronounced for obesity then for diabetes. The pattern in prevalence of diabetes mirrors somewhat the obesity pattern indicating, as argued by others, the possibility that the increase in the prevalence of obesity is the main cause of the increase in the prevalence of diabetes [16]. As both the prevalence of obesity and diabetes have increased markedly in a wide range of socio-demographic and geographically diverse groups, period effects seem an obvious conclusion. It should be noted that, as argued by Reither, period related ‘shifts in social, cultural, economic, or physical environments’, are not totally consistent with important socio-economic differences found for prevalence rates for both obesity and diabetes [3,7,15]. It seems for our results that the story for obesity is centred on females with females having increased age and cohort effects. For diabetes somewhat of a different result is apparent with males more likely to have an age and cohort effect.

Weaknesses of the study included the use of self reported BMI and diabetes. We have previously reported on the bias associated with this form of measurement for BMI with acknowledgment that self report generally over-estimates height, especially for males and older person, and under-estimates weight, especially for women [25]. In addition, much of the literature cited in this manuscript use a variety of statistical techniques and different ways of undertaking APC analyses so direct comparison is difficult. There is no consensus in the literature on the optimal approach to undertake APC analysis [5,30] but we decided on the current analysis because it allows for a non-linear trend. It should also be acknowledged that APC has been criticised as a methodology because of the lack of independence of the age, period and cohort variables from each other with birth cohort = period—age [5,29,31]. In addition, as argued by Lee et al, the rational behind APC analyses are to describe patterns and trends, and as such no statistical significance tests were conducted to prove or disprove hypotheses [23]. It should also be noted that the relatively short time span of the SAMSS data limits period effects to be truly assessed. In addition, the socially desirability of self reported height and weight may have been an issue with excess weight now seen as a sensitive issue. There was a potential bias from survey non-response in this study and this should be seen as a weakness of the study. There is a trend towards lower response rates in all types of population surveys as people protect their privacy, or are overwhelmed by marketing telephone calls or mail outs.

The strengths of the study include the size of the database, and the fact that there was no change in definitions throughout the period of data collection although the increase in diagnosis techniques and promotion makes cases of diabetes more likely to be diagnosed [29]. An additional strength of the study was the established consistency of the data collection methodology over the period of data collection and the short time since completion of data collection. Both of these later points are inherent strengths of a continuous risk factor surveillance system.

By simultaneously considering the effects of age, period and cohort we have provided additional evidence for the value of effective public health interventions. In addition, the analysis provides important considerations, both past and present, when assessing trends in obesity and diabetes and provides a better understanding of the different groups to target for both of these present day public health concerns.
